# Intestinal Obstruction Due to Ingested Olive Pits

**DOI:** 10.7759/cureus.73257

**Published:** 2024-11-07

**Authors:** Helen Bolanaki, George Pappas Gogos, Anastasios J Karayiannakis

**Affiliations:** 1 Second Department of Surgery, School of Medicine, Democritus University of Thrace, Alexandroupolis, GRC

**Keywords:** computed tomography abdomen, foreign body, fruit pit, ileus, intestinal obstruction, small bowel obstruction

## Abstract

Intestinal obstruction after ingestion of foreign bodies is a rare condition. A wide variety of ingested foreign bodies has been reported as a cause of mechanical small bowel obstruction, with ingested fruit pits being rarely reported as causes of intestinal obstruction. Here, we report the case of a 70-year-old female diagnosed with intestinal obstruction due to swallowed olive pits tightly impacted in the distal ileum. A conclusive diagnosis was made preoperatively by computed tomography of the abdomen. After initial conservative treatment, the patient underwent laparotomy because of persistent obstruction. The ingested pits were removed through an enterotomy and the obstruction was successfully resolved.

## Introduction

Intestinal obstruction is a common condition most often occurring after previous abdominal operations with subsequent formation of intraperitoneal adhesions. Ingestion of foreign bodies is another cause of mechanical small bowel obstruction. A wide variety of ingested foreign bodies has been reported as a cause of intestinal obstruction, whereas small bowel obstruction caused by ingested fruit pits is a rare clinical entity [1-3]. Small bowel obstruction due to ingested fruit pits is more likely to occur if there is an undiagnosed intestinal pathology such as tumors or inflammatory bowel disease, whereas, in the normal intestinal tract, it is unusual [4-6].

In this case report, we present a 70-year-old female diagnosed with intestinal obstruction with abdominal computed tomography (CT) revealing ingested fruit pits as the cause of small bowel obstruction.

## Case presentation

A 70-year-old woman presented to the emergency department with abdominal pain and vomiting. She noted a colicky pain over the entire abdomen 48 hours earlier that was followed by vomiting. She had been unable to pass flatus and stool since that time. Her past medical history was unremarkable and there was no history of previous abdominal surgery.

On physical examination, she was afebrile and clinically stable without tachycardia or hypotension. Abdominal examination revealed abdominal distension and mild tenderness on palpation with increased, high-pitched bowel sounds. There were no umbilical, inguinal, or femoral hernias, and no palpable mass was found. Laboratory tests showed a slightly elevated white cell count at 10,980 per mm^3^ with a slight shift toward an increased percentage of neutrophils.

Hemoglobin and hematocrit levels were normal. Serum concentrations of electrolytes, urea, creatinine, and C-reactive protein were within the normal range. There was no elevation in serum lactate level or evidence of metabolic acidosis.

**Figure 1:**
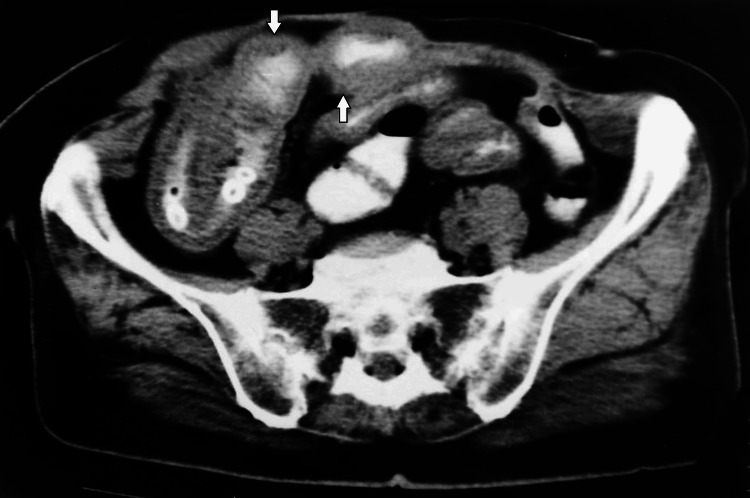
Abdominal computed tomography showing several intraluminal radiopaque foreign bodies in the terminal ileum and thickening and dilatation of the small bowel (arrows).

**Figure 2:**
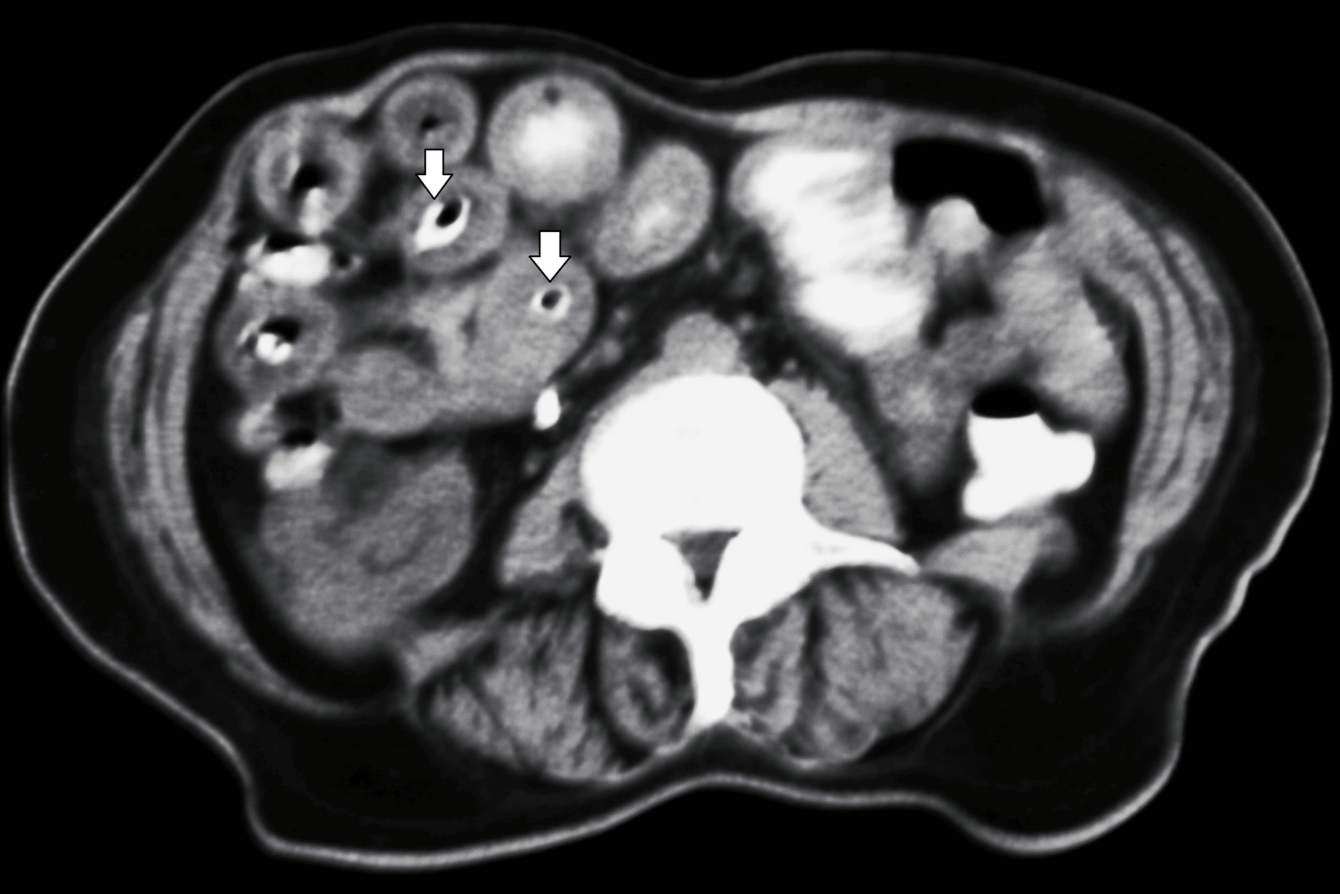
The foreign bodies showing a characteristic concentric “ring-like” appearance consistent with a fruit pit (arrows).

Plain abdominal radiography revealed dilatation of the small bowel and multiple air-fluid levels. Abdominal CT showed thick-walled and dilated intestinal loops and several well-defined, radiopaque, concentric objects in the lumen of the distal ileum (Figures 1, 2). There was no evidence of vascular compromise, perforation, or closed-loop obstruction.

The patient was initially treated conservatively with the presumptive diagnosis of uncomplicated small bowel obstruction due to foreign bodies. The treatment included intravenous fluid administration, nasogastric decompression, and bowel rest. The patient remained under close observation with clinical examination, laboratory testing, and abdominal radiographs performed daily.

Three days later, she underwent an exploratory laparotomy because of persistent obstruction evident by persistent physical signs and abdominal radiography findings. At operation, the small bowel was found grossly dilated with edematous and thickened wall. The foreign bodies were palpated at the terminal ileum. An enterotomy was performed and seven fruit pits were removed. After surgery, the patient admitted to swallowing several whole olives. The fruit flesh was digested over time and only the fruit pits were found during surgery. She had an uneventful postoperative course and was discharged on the fifth postoperative day.

## Discussion

Mechanical small bowel obstruction occurs most often after previous abdominal operations and subsequent formation of intraperitoneal adhesions. Ingested foreign bodies of a wide variety have been reported as another cause of intestinal obstruction. However, small bowel obstruction caused by ingested fruit pits of varied nature is rarely reported in the literature [3-6].

Ingestion of fruit pits either incidentally or intentionally is much more common than anticipated, especially in children, debilitated persons, or psychiatric patients, and is considered a frequent and innocuous event. In most cases, fruit pits pass through the intestinal tract without any symptoms [1]. Intestinal obstruction is a rare complication occurring most commonly in cases of underlying small bowel pathology such as an asymptomatic stricture in Crohn’s disease [4-6]. There have been some reports where the lodged fruit pits resulted in the diagnosis of an asymptomatic tumor or inflammatory bowel disease.

The diagnosis of intestinal obstruction due to ingested fruit pits is not always evident, particularly when a reliable and/or complete history is not available. The symptoms and signs are representative of intestinal obstruction, with vomiting and absence of flatus and stool passage being the main symptoms. Physical examination reveals abdominal distension and increased bowel sounds. Plain abdominal radiography demonstrates small bowel dilatation and air-fluid levels. However, it is difficult to determine the cause of obstruction because fruit pits are of low radio-opacity. Abdominal CT is very useful for correct diagnosis. Typically, there are thick-walled and dilated intestinal loops due to obstruction. The fruit pits are shown as well-defined, radiopaque, concentric objects in the lumen of the small bowel. Other causes such as metallic or plastic foreign bodies, bezoars, gallstones, and enteroliths should be considered, and CT can be helpful in their differentiation [7]. Management of this condition is initially conservative with surgical removal of the foreign body being necessary in case of non-resolving obstruction.

## Conclusions

Small bowel obstruction secondary to ingested fruit pits, although a rare clinical entity, should be considered in cases of intestinal obstruction, especially when complete history is unavailable. Correct preoperative diagnosis can be made by abdominal CT, and surgical removal of the fruit pits should be considered in case of persistent obstruction.
